# Embolic myocardial infarctions look different: a comparison of experimental and fateful embolic lesions

**DOI:** 10.1186/1532-429X-13-S1-P152

**Published:** 2011-02-02

**Authors:** Ralf Wassmuth, Jeanette Schulz-Menger

**Affiliations:** 1 Charite, University Berlin, Berlin, Germany

## Introduction

According to the wavefront-phenomenon myocardial infarction scar should have a larger extent on the subendocardial than the subepicardial surface. This is important for differentiating ischemic from nonischemic lesions in late gadolinium enhancement (LGE) images.

In hypertrophic obstructive cardiomyopathy septal ablation (TASH) with injection of microparticles instead of ethanol represents a controlled model of iatrogenic embolic infarction.

We compared cardiac magnetic resonance (CMR) scar patterns after microparticle-induced TASH with those after clinical embolic infarctions from other sources.

## Methods

18 patients with hypertrophic obstructive cardiomyopathy (13 male, 60±17 years) underwent injection of foam particles into a septal coronary branch. We compared the shape of the resulting myocardial scar with those in 23 consecutive patients (14 male, 55±15 years) with evidence of embolic myocardial infarctions. Embolic infarctions were defined as small single or multiple myocardial scars in conjunction with a normal coronary angiography or a single thrombotic lesion without evidence of general atherosclerosis. A history of embolic events in other vascular territories or the presence of atrial shunts confirmed the diagnosis. Patients with evidence of acute inflammation were excluded. In all patients 3 long axes and a contiguous stack of short axis LGE images were obtained after 0.2 mmol/kg Gd-DTPA in 1.5 a T scanner. In-plane resolution was 1.8 mm/pixel.

Using Osirix 3.3.2., we measured maximal subepicardial and subendocardial extent on LGE images in that short axis slice with the largest extent of the scar. Thereby we differentiated wedge-shaped scars (subepicardial extent > subendocardial extent) from transmural (subepicardial = subendocardial) or subendocardial scars (subepicardial < subendocardial).

## Results

After embolic TASH, infarction scars showed a wedge-shaped pattern in 13/18 cases (72%) with a subendo-subepicardial ratio of 0.8±0.3. In patients with embolic infarctions, 7 cases showed multiple lesions. Wedge-shaped lesions with larger epicardial extent were found in 11, transmural lesions in 10, subendocardial lesions in 2 cases. The mean subendo - subepicardial ratio of embolic infarctions was 0.6±0.1**.**

## Conclusions

Embolic infarctions after septal ablation in HOCM show a wedge-shaped pattern with a larger extent on the epicardial surface, resembling the pattern of other embolic infarctions. This wedge-shaped pattern contradicts the wavefront phenomenon. When differentiating non-ischemic from ischemic lesions in late enhancement images this phenomenon should be taken into account to avoid misclassification of embolic infarction scars as nonischemic lesions.

**Figure 1:**
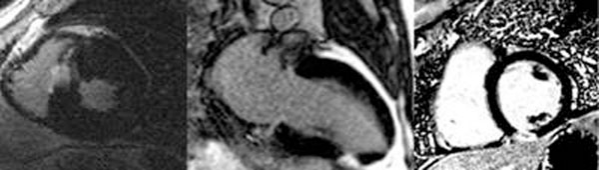
Wedge-shaped scar after embolic TASH in HOCM (left) and 2 different patients (center and right) with embolic infarctions.

